# Laser Sintering of CNT/PZT Composite Film

**DOI:** 10.3390/s23063103

**Published:** 2023-03-14

**Authors:** Yu Sung Chuo, Sina Rezvani, Xavier Michaud, Simon S. Park

**Affiliations:** Mechanical and Manufacturing Engineering, University of Calgary, 2500 University Drive NW, Calgary, AB T2N 1N4, Canada

**Keywords:** laser sintering, PZT, CNT, piezoelectricity, sensors

## Abstract

The discovery of piezoelectricity inspired several sensing applications. For these applications, the thinness and flexibility of the device increase the range of implementations. A thin lead zirconate titanate (PZT) ceramic piezoelectric sensor is advantageous compared with bulk PZT or a polymer when it comes to having minimal impacts on dynamics and high-frequency bandwidth provided by low mass or high stiffness, while satisfying constraints regarding tight spaces. PZT devices have traditionally been thermally sintered inside a furnace and this process consumes large amounts of time and energy. To overcome such challenges, we employed laser sintering of PZT that focused the power onto selected areas of interest. Furthermore, non-equilibrium heating offers the opportunity to use low-melting-point substrates. Additionally, carbon nanotubes (CNTs) were mixed with PZT particles and laser sintered to utilize the high mechanical and thermal properties of CNTs. Laser processing was optimized for the control parameters, raw materials and deposition height. A multi-physics model of laser sintering was created to simulate the processing environment. Sintered films were obtained and electrically poled to enhance the piezoelectric property. The piezoelectric coefficient of laser-sintered PZT increased by approximately 10-fold compared with unsintered PZT. Moreover, CNT/PZT film displayed higher strength compared with PZT film without CNTs after the laser sintering while using less sintering energy. Thus, laser sintering can be effectively used to enhance the piezoelectric and mechanical properties of CNT/PZT films, which can be used in various sensing applications.

## 1. Introduction

In many areas of engineering, including the manufacturing industry, there is growing interest in the monitoring of processes that can automatically provide feedback in order to optimize workflow. Embedding sensors into manufacturing tools and analyzing the resulting data can help processes by reducing production costs, allowing for remote monitoring, and improving efficiency and safety. This can result in adaptable production lines or other services capable of meeting ever-changing needs. Piezoelectric sensors are used due to their high sensitivity, fast response and the high-frequency bandwidth they can provide.

For dynamics measurement, the size and type of sensors can affect the measurement results. For example, thick piezoelectric bulk ceramic sensors affect the dynamics of the system under measurement due to mass loading [1]. Since the bending radius has an inverse relationship to the film thickness [2], a bulk profile would not be flexible and would exert a moment on the system. Meanwhile, polymer sensors have low stiffness and they suffer from creep phenomena [3]. Thin and lightweight ceramic film sensors have minimal influence on the mass and stiffness of the subject of measurement and operate with a wide frequency range, therefore avoiding having a significant impact on dynamics.

Polycrystalline lead zirconate titanate (PZT) is a suitable sensor material, as it is cost-effective compared with single crystals with similar piezoelectric properties, and it provides a higher stiffness and sensitivity than piezoelectric polymers [4]. PZT device manufacturing conventionally involves thermal sintering inside a furnace at more than 800 °C. In this process, the temperature is raised over many hours, and the entire furnace chamber is heated using kilowatts of power. Therefore, the conventional furnace requires a great amount of time and energy consumption and results in high greenhouse gas (GHG) emissions.

When making a film device, a thin substrate can be used to attach the PZT. To this end, a variety of metallic substrates, such as stainless steel, aluminum and tin, along with polymers with conductive coatings, can be considered. However, metallic and polymer substrates are sensitive to high temperatures due to oxidation or melting. Improved flexibility and stretchability of PZT energy harvesters were demonstrated on low melting point substrates, such as poly(ethylene terephthalate) [5,6] and poly(dimethylsiloxane) [7]. Due to the thermal sintering temperature for PZT being typically higher than the substrate melting/oxidizing temperature, these devices were realized using complex film transfer processes [2].

Laser sintering makes use of a high-intensity laser beam to scan the film of interest in continuous or pulsed modes. Laser energy is absorbed by the film to introduce atomic diffusion. Due to the localized energy, the process could require much less time and energy consumption, using just a small fraction compared with conventional furnace sintering. The processing time for laser sintering would range from seconds to minutes, which is primarily dependent on the sample size and scanning pattern. Laser sintering could bring other benefits as well since the energy and penetration depth can be tuned by the intensity, wavelength of the laser and the absorption properties of the material [8]. Laser sintering may avoid thermal damage to the temperature-sensitive substrate because the interface temperature of films is tunable by controlling the absorbed laser intensity. Moreover, it could allow a low-temperature substrate to be compatible with high-temperature sintering, as the laser radiation and heat conduction are one-directional and of short duration. The possibility of directly sintering the PZT–substrate assembly without damage would ease manufacturing and decrease the production time. Moreover, the adaptability of laser processing is further enhanced by using a computer-controlled scanning geometry [9]. Therefore, affirmative sintering results could reduce manufacturing costs, spurring sensor development and adoption.

Although PZT ceramics have good piezoelectric properties, they are fragile with low fracture toughness, which limits their durability. To improve the strength, nanotubes can be added to the PZT matrix to create a composite. Carbon nanotubes (CNTs) are a promising option for this purpose because they have high strength-to-weight ratios [10]. Thus, the desirable properties of the high sensitivity of PZT and the high strength of the reinforcement are combined. CNTs have high absorption for a large portion of the light spectrum. They also have high thermal conductivity [11,12,13]. Hence, adding CNTs could increase the absorption of electromagnetic radiation, as well as create a more uniform temperature distribution due to their high thermal conductivity. It was reported that the addition of CNTs in a microwave sintering setup increased the heating rate [14]. Similar benefits regarding CNT heating can be realized in a laser setup as well. Cox et al. reported that graphite, which has the same oxidation mechanism as CNTs, oxidizes at rates of 25.2–38.1 µg/s when the temperature is 900–1200 °C [15]. Hence, in furnace sintering conditions, the CNTs may completely burn off due to the prolonged high-temperature process. The short-duration laser sintering could exploit the benefits of CNTs as a sintering aid and reinforcement additive while the oxidation has not reached completion.

Laser processing on a high-temperature compatible substrate with oxide layer construction made from chemical vapour deposition [8] and RF sputtering [16] was previously attempted. Laser sintering on a simpler substrate is quite limited. The processing usually applies the methods of sol-gel processing [17], vapourization [18,19] and melting [20], which require crystallization. These film and substrate preparation methods are more complex, take more time to deposit, or have higher equipment and fabrication costs than tape casting. Previous laser sintering PZT powder is very scarce and without characterization of the resulting piezoelectric coefficient. The method of tape casting, which is suitable for large-volume and low-cost processing of PZT film, has not been attempted for use with laser sintering. The laser types generally employed were CO_2_ in the medium IR range of 10,600 nm and KrF and XeCl excimer in the medium UV range of 248–308 nm. The use of Nd:YAG in the near IR range of 1064 nm is limited for ceramic processing. Furthermore, while laser processing was attempted on the composites of SiO_2_/PZT and PVDF/PZT [21], it was not attempted on CNT/PZT composite.

This research aimed to develop a cost-effective thin piezoceramic manufacturing for sensing applications using laser sintering and compared the results with furnace sintering. To address the brittleness and low thermal conduction of PZT, CNTs were added to increase mechanical properties and to act as a sintering aid stemming from the increased absorption of electromagnetic radiation and thermal conductivity, thereby improving the sintering efficiency. To improve understanding of the laser interaction inside the material, the temperatures of the top and bottom surfaces of the film during the laser sintering were verified via modelling of the laser heating mechanism, which included the experimental parameters and conditions. This was used to predict laser heating effects and to assess suitability with the low-temperature substrate. Optimization of the laser sintering parameters was done by alternating the control parameters and with the aid of modelling. Finally, the sintered sample that utilized laser processing was used to make a force sensor demonstration and compare it with a reference commercial sensor.

## 2. Materials and Methods

**Figure 1:**
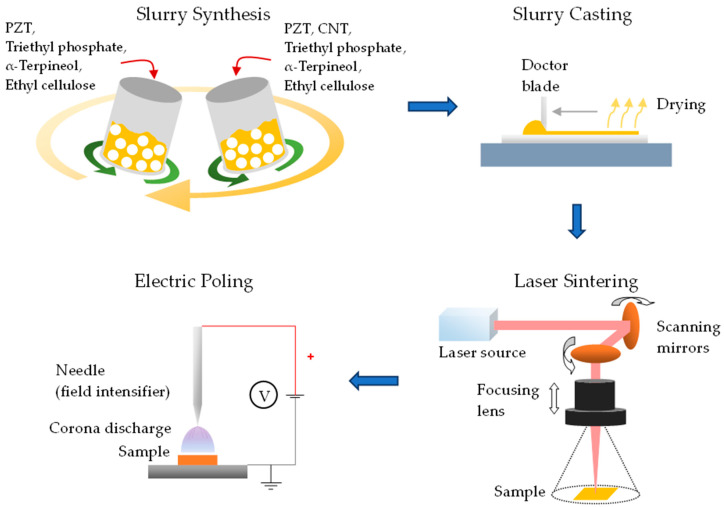
Experimental procedures associated with laser sintering.

An outline of the experiments is shown in Figure 1. Samples were prepared through stages of ink slurry or particle synthesis, forming via slurry casting or particle pressing, sintering via laser or conventional furnace and post-sintering treatment of poling. The figure illustrates the steps for the PZT film fabrication. The samples sintered from the laser and furnace were used for comparison.

### 2.1. Forming before Sintering

#### 2.1.1. Slurry Synthesis and Casting

To prepare the ink for casting via doctor blading, jet printing or screen printing, the mixtures were prepared by combining PZT powder, solvent, binder, dispersant and/or plasticizer [2,22]. A sintering aid was used in some samples to facilitate the initial stages of sintering and reduce the temperature requirements [23]. The solvent acted as a carrier of the solution and was used to dissolve the binder, dispersant or plasticizer, producing a form suitable for casting. The binder was used to promote the adhesion of the ink slurry when cast on a substrate. The dispersant was used to ensure a uniform dispersion to prevent the clustering of the particles. The plasticizer was added to the ink slurry to make the casted part softer and more flexible without cracking, which made the handling of the green sheet easier.

The ink slurry used for doctor blading was made with the formulation in Table 1. The PZT powder (Tcera Co., Kaohsiung, Taiwan) was a low-sintering-temperature soft PZT. It has a theoretical density of 7.9 g/cm^3^ and a sintering temperature between 900 and 1000 °C. The particles had an average diameter of 0.54 μm. The PZT powder was mixed in isopropyl alcohol, in which the triethyl phosphate (Sigma Aldrich Chemical Co., Oakville, ON, Canada) dispersant was dissolved to obtain a coating on the powder surface. The suspension was placed in a planetary centrifugal mixer (TMAX XXH300S) and mixed for 2 h, and it was subsequently dried at 40 °C for 24 h. A solution of ethyl cellulose (Thermo Fisher Scientific Co., Waltham, MA, USA) binder in α-terpineol (Sigma Aldrich Chemical Co.) solvent was made via magnetic stirring on a hot plate at 100 °C for 5 h. Then, the dried PZT powder was dispersed in the prepared solution and mixed in the planetary centrifugal mixer for 2 h in order to homogenize it.

Achieving good dispersion of CNTs in a ceramic matrix is one of the main challenges of fabricating a CNT-reinforced ceramic composite [12]. Functionalizing CNTs with a carboxyl group (COOH) can alleviate the problems, as the added polarity improves the dispersion and bond strength [24]. For the composite samples, COOH-functionalized CNTs with an outer diameter of 30–50 nm, inner diameter of 5–10 nm and length of 10–20 μm (Cheap Tubes Inc., Grafton, VT, USA) were first ultrasonically dispersed in isopropyl alcohol for 1 h, followed by the addition of the PZT powder and the same procedure as described above was carried out to prepare the slurry. The ratio was designed so that after solvent drying, there would be 0.5 wt. % of CNT. However, since the density of the CNT is 2100 kg/m^3^ (Cheap Tubes Inc.), which is much lower compared to the density of the PZT, i.e., 7900 kg/m^3^ (Tcera Co.), the volume ratio of the CNT in the dried film was 1.86%.

A thin layer of the ink slurry was spread on an indium tin oxide (ITO) coated soda-lime glass using the doctor blade method. The thickness of the slurry was 132 μm, which was the thickness of the tape layer (3M Scotch 2020) used to make the mask. The casted sample was dried in a vacuum oven at 40 °C for 24 h. After drying, the thickness decreased to 45 μm.

#### 2.1.2. Particle Pressing

To prepare pellets for furnace sintering, a powder mixture containing 99% PZT and 1% polyvinyl alcohol (PVA) was prepared. It was synthesized by dissolving PVA particles in deionized water using magnetic stirring at 150 rpm on a hot plate with a temperature of 130 °C. Then, the solution was added to PZT particles and the contents were mixed by employing a pestle and mortar. After drying the mixture on a hot plate at 80 °C, a 200-mesh sieve was used to obtain a uniform particle size distribution. Then, 4 g of powder was placed inside a cylindrical mould with a bore of 20 mm. The mould was pressed uniaxially in a manual hydraulic machine to a pressure of 200 MPa to fabricate the pellets [25].

### 2.2. Sintering

#### 2.2.1. Laser Sintering

**Figure 2:**
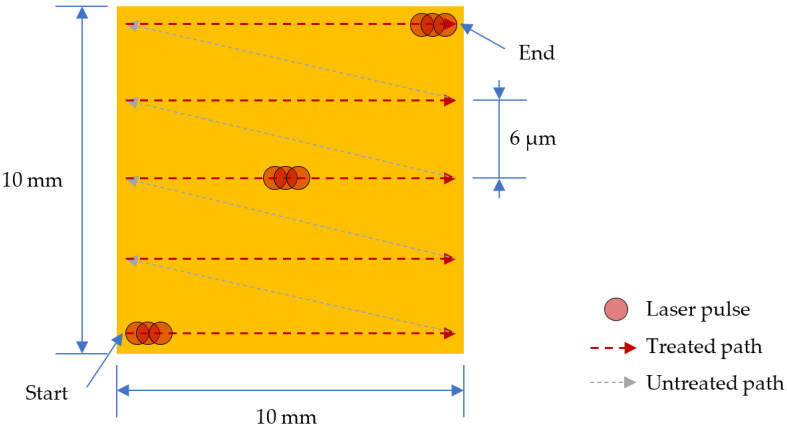
Laser beam scanning pattern.

The laser setup utilized light wave irradiance to impart energy to the workpiece. A hot plate was used to gradually preheat the sample from room temperature to 500 °C in order to reduce the thermal gradient between the top and bottom surfaces and reduce the crack occurrence due to thermal stress. The organic binder phase is partially eliminated during preheating step as well, which changes the density. For the composite sample, the preheating temperature was lower at 420 °C in order to limit the amount of CNT oxidation during the preheating step. A Nd:YAG fiber Q-switched laser machine (IEHK Enterprises FM20W laser engraver) with a wavelength of 1064 nm and a maximum power of 20 W was used to conduct the sintering on PZT ceramics and CNT/PZT composites. The frequency of pulses was set at 30 kHz, and each pulse had a duration of 100 ns. The laser beam diameter varied with the distance between the focusing lens and the sample and could achieve a small size of 70 µm at the focused position. The laser beam moved across the sample, as depicted in Figure 2. Starting from the bottom, it sintered from left to right, as indicated by the red path. Afterwards, the beam turned off and moved to the next starting point, as indicated by the grey path, and the beam activated again to continue the process. After sintering, the hot plate was cooled down gradually to room temperature.

Due to the highly localized energy, heating using a laser is more prone to produce cracks or other forms of damage to the film; hence it is important to control the laser energy per area and time. The laser sintering parameters needed to undergo optimization in order to avoid adverse effects. The most crucial parameter that directly affects the laser energy is the power. In this study, the power parameter changed from 40% to 100% of the maximum power using steps of 20%. The laser path spacing, speed and number of repetitions were kept constant at 6 µm, 1050 mm/s and 1, respectively. The path spacing and the speed were chosen to provide sufficient overlap between each scan path and between each pulse, which aimed to provide consistent sintering and minimize sintering variation due to the scanning pattern. The laser lens moved up from its focal point by 30 mm to broaden the laser beam, which had the effect of increasing the area for energy distribution and allowed for using higher power.

#### 2.2.2. Furnace Sintering

For conventional furnace (Knights Furnace KMT10-9) sintering, the temperature was ramped at 3 °C/min until it reached 540 °C, where it was held for 1 h for complete binder burnout. Afterwards, the temperature was ramped at 1.4 °C/min until it reached 950 °C, where it was held for 2 h for the complete sintering. Then, the sample was cooled down at 1.7 °C/min until it reached room temperature.

Fabrication of the laser-sintered sample took 1.5 h of preheating and 10 s of laser sintering. In contrast, furnace sintering of the same part would take many hours more to reach the sintering temperature and would need to include a film transfer process as well. The localized heating allowed the laser technique to attain substantial time and energy savings. Moreover, the precise definition of the laser beam location allowed it to sinter in a shape or size conducive to the application.

### 2.3. Electric Poling

**Figure 3:**
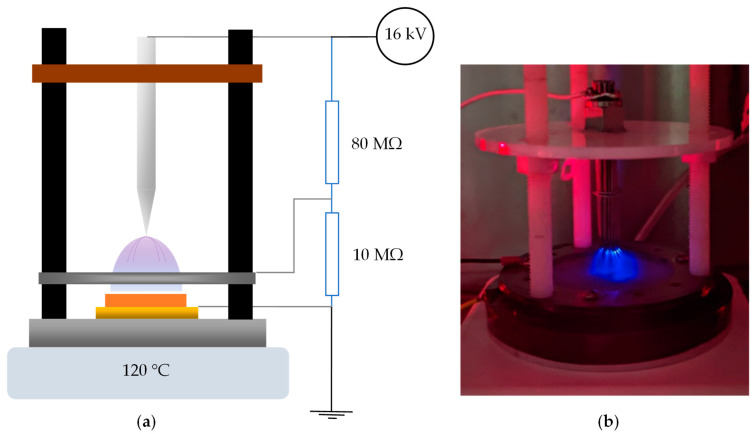
(**a**) Corona poling schematic; (**b**) photo of the corona poling process.

The samples were poled using a corona discharge setup to align the dipoles inside them. The process is displayed in Figure 3. This was carried out at 120 °C for 2 h with a DC voltage of 16 kV applied to a set of needles, which acted as field intensifiers. This caused the ionization of surrounding gas molecules [26]. The sample was placed on a grounded copper plate, which was connected to the bottom electrode of the sample. An electric field created by the electric charge from the needles was sprayed onto the top surface of the sample. A metal mesh was inserted between the needles and the sample to limit the intensity of the corona discharge. A voltage divider provided 11% of the supplied voltage to the mesh. The temperature was cooled while keeping the applied electric field, after which the field was removed. The samples were set aside to allow for one day of aging.

A wide-range $d_{33}$ meter (APC YE2730A) was used to measure the piezoelectric coefficient of the unsintered and sintered samples. The piezoelectric coefficient $d_{33}$ can be defined as the ratio of electric charges (Q) generated in response to the applied force (F) when both the applied force and generated charge are in the poling direction.

An optical profilometer (KLA Zeta 20) and a scanning electron microscope (Phenom XPro) with a built-in energy-dispersive X-ray spectroscope (EDXS) were used to observe the change in surface roughness and morphology on the green and sintered samples, as well as the elemental composition. X-ray diffraction (XRD) (Bruker D8 Advance Eco) was used to examine the crystallinity and any possible phase change with the sintering, as well as when adding CNTs. Mechanical testing was done using a microindenter (Bruker Hysitron TI Premier).

## 3. Modelling of Laser Sintering

The capability to obtain an accurate measurement of the laser heating process rarely occurs due to limitations in the commonly available temperature measurement technologies. A heat transfer simulation was conducted in order to estimate the temperature from laser sintering. It allowed for a large variety of configurations, such as substrate material, thickness and processing parameters to be modelled. The temperature increase had a positive relationship with the sintering density and grain changes.

**Figure 4:**
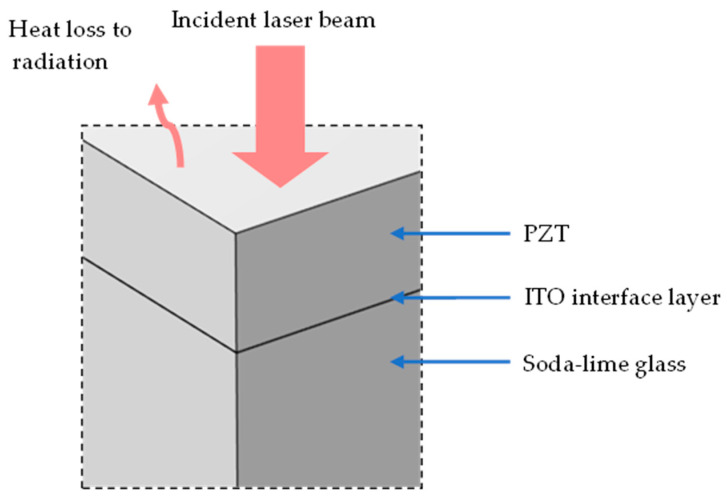
Three-dimensional model geometry used in the heat transfer simulation of laser sintering.

Figure 4 shows the setup that illustrates the modelling and boundary conditions of the temperature simulation.

Typically, the time dependence of the energy distribution of the pulsed laser exhibits a Gaussian shape [8]. Therefore, the time distribution of the laser beam intensity ($I\left(t\right)$) follows the following trend:

(1) $$I\left(t\right)=\frac{E}{\left(S\cdot \tau \right)}e^{-4ln2\frac{{\left(t-\tau \right)}^{2}}{\tau^{2}}}$$

where E is the applied laser energy, S is the surface area, τ is the pulse width and t is time.

The Beer–Lambert law is used to model the attenuation of a laser as it travels through the sample. The PZT and substrate layers are the absorbing media. The laser-driven heat source can be described using the following equation [8]:

(2) $$Q_{r}=I(t)\cdot (1-R)\frac{e^{\frac{{-z}_{d}}{\delta_{\alpha }}}}{\delta_{\alpha }}$$

where R is the reflectance (Equation (4)), $z_{d}$ is the depth and $\delta_{\alpha }$ is the penetration depth (Equation (5)). The complex index of refraction can be stated as follows [27]:

(3) $$\tilde{n}=n-ik$$

where n and k are the refractive index and extinction coefficient, respectively. If the value of the extinction coefficient is very small relative to the refractive index, as in the case of most non-metals, it may be omitted in the calculation of reflectance [27,28]:

(4) $$R={\left(\frac{n_{t}-n_{i}}{n_{t}+n_{i}}\right)}^{2}$$

where $n_{t}$ and $n_{i}$ are refractive indices of the transmitting and incident media, respectively.

The penetration depth in Equation (2) is obtained from the extinction coefficient [27]:

(5) $$\delta_{\alpha }=\frac{1}{\alpha }=\frac{c}{2\omega k}=\frac{4\pi k}{\lambda }$$

where α is the optical absorption coefficient, ω is the angular frequency, c is the speed of light and λ is the laser wavelength.

The heat flux from the laser beam on the PZT surface was modelled as a Gaussian distribution according to the following equation:

(6) $$\phi =\frac{2\cdot P_{laser}}{\pi \cdot r_{spot}^{2}}e^{-\frac{2\cdot r_{focus}^{2}}{r_{spot}^{2}}}$$

where $P_{laser}$ is the power of the laser, $r_{spot}$ is the radius of the laser spot and $r_{focus}$ is the distance from the point of the beam centre. Thus, as the distance away from the beam centre increases, the heat flux reduces in the Gaussian distribution.

The area of the beam on the PZT surface is calculated using Equations (7)–(10). The diameter of the laser beam (2w) is defined by the boundary where the intensity drops to $1/e^{2}$ of the maximum intensity. The laser beam passes through a field lens and converges into a focused spot. A smaller spot size results in higher intensity. The spot size at the beam waist ($2w_{0}$), namely, the focused position, can be calculated by [29]:

(7) $$2w_{0}=1.83\frac{\lambda f}{D}$$

where f is the effective focal length of the lens and D is the incident beam diameter at $1/e^{2}$ intensity before entering the lens. The constant (1.83) is related to pupil illumination and the degree of input interception for a Gaussian beam with $1/e^{2}$ diameter. As the position moves from the beam waist, the size of beam begins to diverge, which can be calculated using [30]

(8) $$w(z)=w_{0}\sqrt{1+{\left(\frac{M^{2}\lambda z_{w}}{\pi w_{0}^{2}}\right)}^{2}}$$

where $z_{w}$ is the distance propagated from the beam waist and $M^{2}$ is the beam quality factor, which represents the degree of variation from an ideal Gaussian beam. The relationship between the $1/e^{2}$ width and the full width at half maximum (H) is [31]

(9) $$2w=\frac{\sqrt{2}H}{\sqrt{ln2}}$$

The relationship between the full width at half maximum (H) and the standard deviation σ of the normal distribution is [32]

(10) $$H=2\sqrt{2ln2}\sigma$$

The heat transfer equations are coupled to the radiation in the multiphysics model to obtain the temperature variation. Heat conduction happens in the PZT layer, as well as from the PZT layer to the adjacent layer. The governing heat conduction equation is

(11) $$Q_{r}=\rho C_{p}\frac{\partial T}{\partial t}-\nabla \cdot \left(k_{t}\nabla T\right)$$

where $Q_{r}$ is the heat generated from the absorbed laser energy [33], ρ is the material density, $C_{p}$ is the specific heat capacity and $k_{t}$ is the thermal conductivity. Surface-to-ambient radiation heat flux is modelled on the top boundary. It is described by the following equation:

(12) $$\Phi_{q}=\varepsilon \sigma (T_{a}^{4}-T_{b}^{4})$$

where ε is the emissivity, σ is the Stefan–Boltzmann constant, $T_{a}$ is ambient temperature and $T_{b}$ is the sample temperature.

The material properties of the PZT, ITO and soda-lime glass were entered as given in Table 2. The PZT powder used in this study contained an excess amount of lead, which increased the laser absorption. As the temperature rose to a high level, lead loss reduced the absorption. Hence, at low temperatures, the property of metal-doped PZT was used for reference [34], and at high temperatures, pure PZT was modelled due to lead loss [35,36]. The density was also modelled as temperature dependent, where it started at a level of an unsintered ceramic film. As the temperature reached the melting point, the density changed to the level of a theoretical solid. The reflectance and penetration depth were derived from the complex refractive index. The sample was surrounded by ambient air, which has a refractive index (n) of 1 [37]. Preheating before sintering is included in the model, which was also done in the experiment to limit the thermal shock from the laser heating of the ceramic.

The laser setup properties were entered as shown in Table 3. The other coefficients of penetration depth and beam size distribution are derived from the wavelength, focus length, beam diameter and beam quality factor.

**Figure 5:**
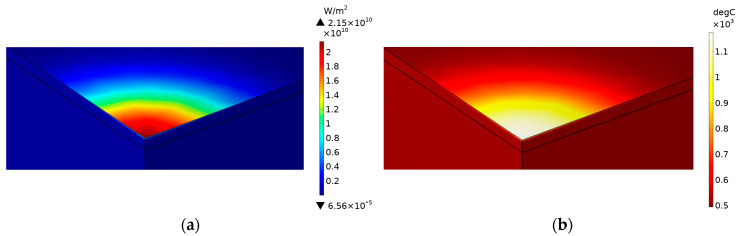
Changes during one pulse. (**a**) Intensity; (**b**) temperature.

**Figure 6:**
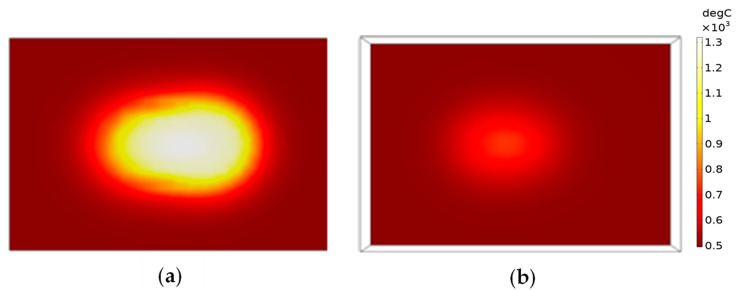
Temperature changes during one area scan. (**a**) Top surface; (**b**) bottom surface.

The model used numerical computations in Comsol^TM^ 6.0 by coupling the multiphysics of radiation and heat transfer using the above conditions, equations and input properties. A lens distance of 30 mm offset from the focused position and full power were simulated. Figure 5a illustrates the change in laser intensity as it penetrates and attenuates in the PZT during one pulse. Figure 5b illustrates the change in temperature due to the absorbed laser during one pulse, which reached 1185 °C. After the pulse, heat conduction continued to adjacent areas. To model area sintering, the same scanning pattern shown in Figure 2 was implemented. Thus, the model incorporated the compounding effects of pulses and line scans in one area scan. The temperature profiles are shown in Figure 6. The top surface reached 1310 °C and the bottom surface reached 780 °C. During each pulse, the sample heated up in an extremely short time of nanoseconds. The high temperature reached on the top region allowed for rapid sintering. Due to the overlap of hot regions between each pulse, the temperature successively built up in the area scanning simulation and was higher than that from a single pulse. The overlap also produced consistent sintering on the sample during the laser scanning. The pulse build-up also meant that the sintering could be influenced by the speed of scanning, which affected the laser beam contact time. A slower speed increased the contact time with the sample surface and resulted in a higher temperature and vice versa. Therefore, this parameter was included in the optimization trials, which produced sufficient overlap for sintering consistency, but did not overheat the sample surface. Heat conduction allowed for some temperature increase to the bottom interface as well. Due to the attenuation, as well as the low thermal conductivity of PZT, there was a difference in the top and bottom surfaces, and the substrate remained at a low temperature to avoid thermal damage.

The effects of adding CNTs were also modelled. The material properties of CNTs were found in published sources [52,53]. The rule of mixtures was used to predict the properties of the composite, as shown in Table 4.

Given the same laser energy input, the sample with CNTs exhibited a higher temperature rise to 1381 °C compared with 1310 °C without CNTs. This was due to the very high absorption possessed by CNTs, which converts larger amounts of energy into heat. Furthermore, the very high heat conduction to the PZT particles could assist in the distribution of sintering energy, and the sintering consistency during the area scanning was achieved as well. Thus, the CNTs could act as a sintering aid, and the sample containing CNTs could require less energy for sintering. However, the absorption characteristics of CNTs also meant that the laser beam attenuated faster, and the substrate would not be damaged either. If a significantly higher concentration of CNTs was used, the temperature rise would have been much higher beyond the peak seen with 0.5% CNTs and could have caused ablation of the sample. With the high concentration of energy density in the areas of irradiation, the laser heating models predicted that the directed energy of the laser method achieved higher temperatures in the PZT and CNT/PZT films compared with the furnace. Since the rate of sintering has an exponential relationship to the temperature, rapid sintering was enabled.

In order to verify the simulation method, a pyrometer (Micro-Epsilon CTRM-2H1SF100-C3) was used to measure the temperature change in the PZT sample. The simulated temperature was 3% higher than the pyrometer-recorded temperature. Contributions to the error may include approximating material properties of the PZT compact and gas flow, which were not modelled in the simulation, and the pyrometer response rate, which was 1 ms.

## 4. Results and Discussion

The effectiveness of laser processing was assessed based on the piezoelectric, microscopic, spectroscopic and mechanical properties. The characteristics gave an indication of the sensing implementation. The properties were compared based on the sintering method and composition. Moreover, a laser-sintered sample with a low-melting-point substrate was tested in a force-sensing experiment to extract the signal for measurement as verification. In the sections below, the laser-sintered samples were made using 100% power, unless otherwise specified.

### 4.1. Piezoelectric Property

The piezoelectric coefficient ($d_{33}$) of the samples is displayed in Table 5. The laser-sintered PZT had a lower $d_{33}$ compared with the furnace-sintered PZT, which may be explained by two factors: the casting and sintering uniformity. First, it should be noted that PZT film has lower piezoelectric properties compared with bulk PZT [54]. The effects of film processing do not employ a pre-sintering densification technique, such as hydraulic pressing. Even though it may be possible to perform pressing on a PZT film to densify it, the operating pressure might be lower compared with bulk processing. Moreover, a lower electrical field is usually used to pole thin films, as any defect on these samples may cause sparks at the high electric fields. The $d_{33}$ values of PZT thin films are commonly found in the range of 60–130 pC/N [55], and that of a commercially mass-produced PZT film sensor (Huaban Co., Shenzhen, Guangdong, China) is 130 pC/N, which is lower than the bulk value of 420 pC/N. The second factor for the reduction in $d_{33}$ of the sample was the laser technique. The laser-sintered sample received high-intensity energy on the surface, which benefited the rapid sintering and may have been a contributing factor to the $d_{33}$ in the region near the top. However, the sintering activity may have been slower further down the depth of the film, which could possess a smaller grain growth. However, $d_{33}$ was significantly higher in the sintered state compared with the unsintered state, indicating that the laser sintering treatment enhanced the piezoelectric property. Furthermore, it was also higher than piezoelectric polymers, which have an average of approximately 18~30 pC/N [56]. The value for the composite with CNTs showed a decrease compared with the laser-sintered PZT. The CNT oxidation may have created surface defects on the composite sample, such as uneven surfaces or voids, which could have adversely affected the poling performance by decreasing the insulating property. CNTs increased the temperature of the composite during laser processing, which was a contributing factor to the sintering. However, due to the impurity content, the CNT oxidation may leave behind a small amount of ash, which inhibits density increase. For any remaining CNTs that did not oxidize, they would not possess polarization, in contrast to PZT. These effects may combine to result in a lower piezoelectric property. Nevertheless, the composite retained a sensitivity that was easily utilizable.

A comparative study of the $d_{33}$ variation with the laser sintering parameter of power was conducted on the PZT, with different samples sintered from 40% to 100% power and then electrically poled. The trend is displayed in Table 6. A higher power created more sintering due to a higher temperature increase. This increased the diffusion rates, closing the pores and growing the PZT grains, which improved polarization. As the laser beam exited the focusing lens, it began to converge. The closer the lens distance was to its focused position, the smaller the beam diameter, which made the laser energy more tightly concentrated, resulting in higher intensity. The distance from the focusing point was selected so that over-sintering due to the high intensity would not occur, even at 100% power. If the intensity was too high, an excessive temperature rise would have resulted in over-sintering, and damage to the sample in the form of altering the crystal structure or too much lead loss, high diffusion of elements through the substrate, ablation of the PZT or melting of the substrate, and thus, the piezoelectric property would significantly degrade.

### 4.2. Microscopic Properties

The measured surface roughness of the samples is displayed in Table 7 in terms of the average peak-to-valley distance (Sz). The samples from the laser sintering had higher Sz. The intense light imparted highly concentrated energy onto the samples over a relatively short duration. This caused higher thermal gradients within the samples, as evidenced by the heat transfer simulations, and may have caused the surface roughness to increase due to the variation in shrinkage of the sample. The sample with CNTs had a higher value compared with the one without, which was indicative of CNT oxidation on the surface. CNTs located on the surface or close to the surface of the samples are very prone to oxidation. The oxidation of these nanotubes caused some voids, which increased the surface roughness of the samples. Moreover, the PZT particles that directly received heat conduction from CNTs could show faster merging behaviour and create a variation in shrinkage. These might be contributing reasons for the increase in the peak-to-valley roughness measurements. A higher roughness negatively affects the contact area when applying electrodes; therefore, the corona poling approach was evidenced to be more suitable than contact poling.

**Figure 7:**
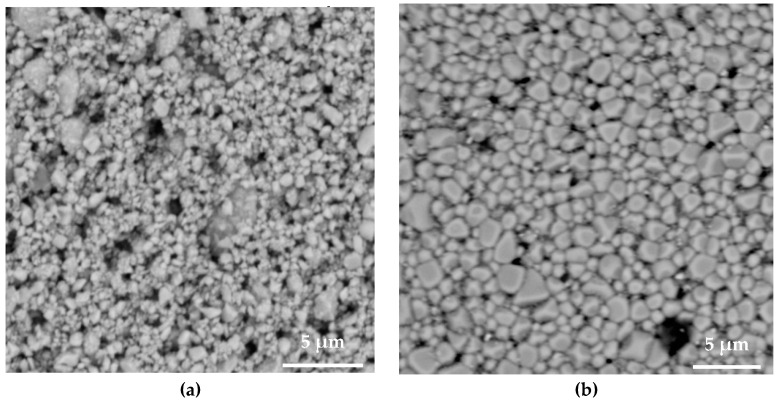

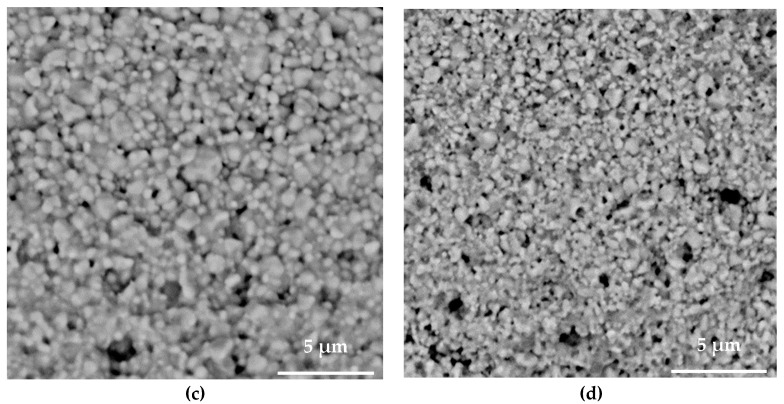
SEM images of (**a**) unsintered PZT, (**b**) furnace-sintered PZT, (**c**) laser-sintered PZT and (**d**) laser-sintered CNT/PZT.

The results of the SEM of sintered surfaces are shown in Figure 7, which compares the green part, furnace-sintered PZT, laser-sintered PZT and CNT/PZT. All sintering methods produced necks between particles. This led to particle merging, grain growth and pore reduction. These changes benefited the enhancement of the piezoelectric property. This was also conducive to higher strength. Furnace-sintered PZT had the most uniform microstructure, which yielded the highest $d_{33}$ measurement. Laser sintering on PZT achieved microstructural changes in a much shorter time than traditional furnace sintering and with lower energy. Although the energy consumption was smaller, the rate of necking created by the intense light wave irradiation was much higher, which allowed the PZT particles to merge significantly during laser sintering. The closing of pores seen on the film surface supported a significant increase in the piezoelectric property, although the $d_{33}$ measurement revealed that the laser-sintered PZT had a lower piezoelectric coefficient than the furnace-sintered PZT. This implies that the rate of laser sintering was lower in the region close to the substrate. The CNT/PZT also displayed large regions of fused particles on the surface and pore size reduction relative to the unsintered state. Although the CNTs’ absorption characteristic caused a higher temperature in laser sintering, the CNTs situated at the grain boundaries between particles may have inhibited the complete fusing action to a degree. Furthermore, the surface was rougher compared with the laser-sintered PZT, as was confirmed by the profilometry measurements as well, which may have been due to some oxidation effect of the CNTs. These outcomes from the CNTs could affect the poling performance, which in turn ended up with a lower piezoelectric coefficient compared with the laser-sintered PZT. Overall, laser sintering imparted changes in the microstructural and piezoelectric properties with a faster turnaround while using lower power compared with the furnace.

**Figure 8:**
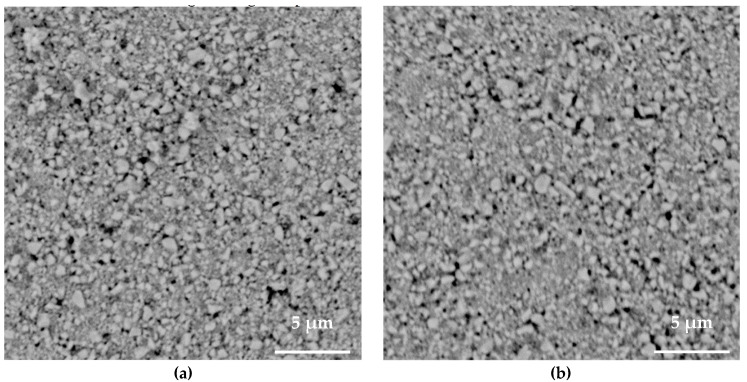

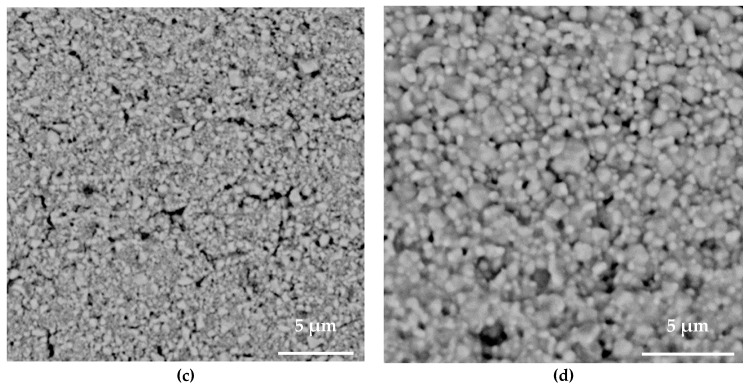
SEM images of PZT laser sintered using (**a**) 40%, (**b**) 60%, (**c**) 80% and (**d**) 100% power.

Figure 8 shows the effect of the laser power on the sintered morphology. The power varied from 40% to 100%. At 40% power, necking between particles occurred, and the pores were smaller relative to the unsintered state. With each successive power increase, the peak temperature rose and the particle merging and grain growth became increasingly visible, resulting in an increase to a substantially higher piezoelectric coefficient at 100% power. The lens distance of 30 mm upwards from the focused position was chosen in order to broaden the laser beam. This had the effect of increasing the area for energy distribution, which decreased the deposited laser intensity on the surface. This technique of moving the field lens to bring the laser–PZT interface out of the focused position allowed for utilizing the highest power without over-sintering/damage.

**Figure 9:**
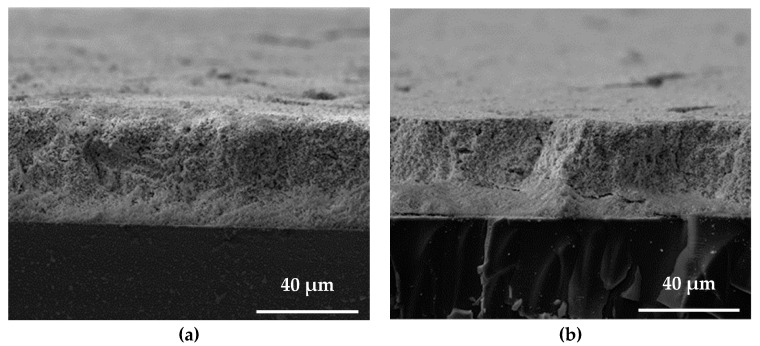
SEM images along the thicknesses of laser-sintered (**a**) PZT and (**b**) CNT/PZT.

To check the sintering results along the transverse cross-section, SEM images were taken along the thicknesses of the samples. The images are shown in Figure 9. The lower region near the substrate in the laser-sintered samples had a visibly different appearance compared with the upper region. The region near the top surface showed larger necking and fusing behaviour. In the lower region away from the top surface, there may have been less sintering, leading to a different appearance. A reason for this could be that there was a temperature gradient from the laser method, corroborating with the laser sintering model. A similar trend was observed for the composite sample.

### 4.3. Spectroscopic and Crystallographic Properties

**Figure 10:**
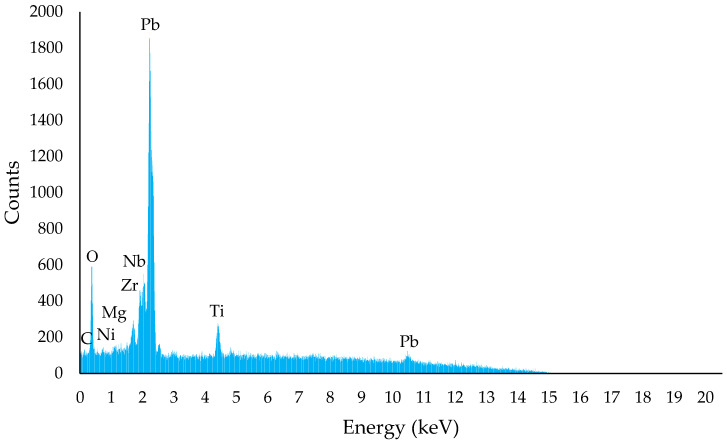
EDXS spectrum of unsintered PZT film.

Energy-dispersive X-ray spectroscopy (EDXS) spectrum of the unsintered PZT film is shown in Figure 10. The elemental ratio provided by the EDXS equipment revealed that the ratio of Zr to Ti was 51% to 49%. This ratio was very close to the morphotropic phase boundary, namely, Pb(Zr_0.52_Ti_0.48_)O_3_, giving it a high charge-carrying capacity [57]. The ratio of Pb was not stoichiometric; there was an excess of Pb in the form of PbO. Liquid PbO changed the sintering medium from a solid state to a liquid state, creating a faster sintering rate and lowering the sintering temperature from 1250 °C to 900~950 °C. Moreover, the EDXS spectrum presents other elements not found in pure PZT, such as Nb, Ni and Mg, which may have been impurity byproducts of the calcination process to manufacture the PZT powder. These trace elements could also assist the laser-sintering process by increasing the light energy absorption.

**Figure 11:**
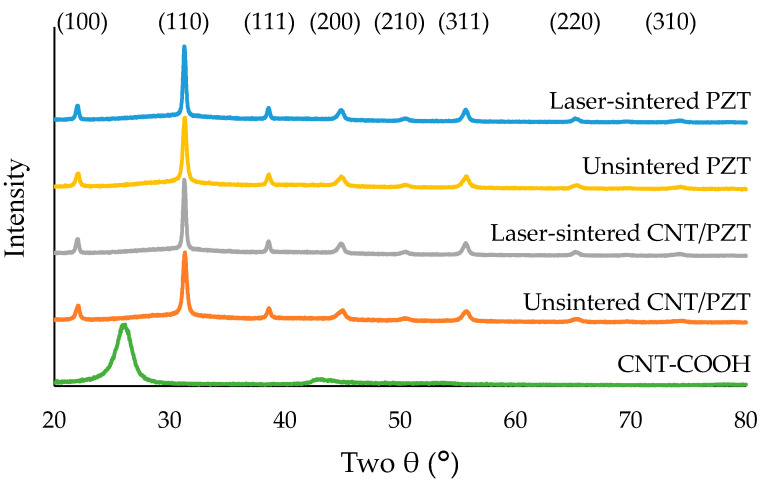
XRD spectra of the laser-sintered and unsintered PZT and CNT/PZT, as well as CNT-COOH.

The XRD spectra of the laser-sintered and unsintered PZT and CNT/PZT, as well as the pure CNTs, are displayed in Figure 11. The laser-sintered and unsintered films had a perovskite crystalline structure with matching peaks at the indicated crystallographic directions [58]. The laser heating did not cause a degradation of the crystal structure, and the well-defined peaks were sustained. When there is an excessive lead loss, this creates a pyrochlore-type phase, which negatively impacts the piezoelectric property. The rapid sintering prevented excessive lead loss from the sample, including on the top surface which had the most intense heating effect. This presents an advantage compared with furnace sintering, where prolonged heating sometimes produces a large pyrochlore phase [59]. The CNTs had significantly different peaks, but due to their low concentration, the composite sample shared the same matching peaks with the PZT perovskite and had the same spectrum. Hence, the laser-sintered films possessed a high piezoelectric property.

### 4.4. Mechanical Properties

**Figure 12:**
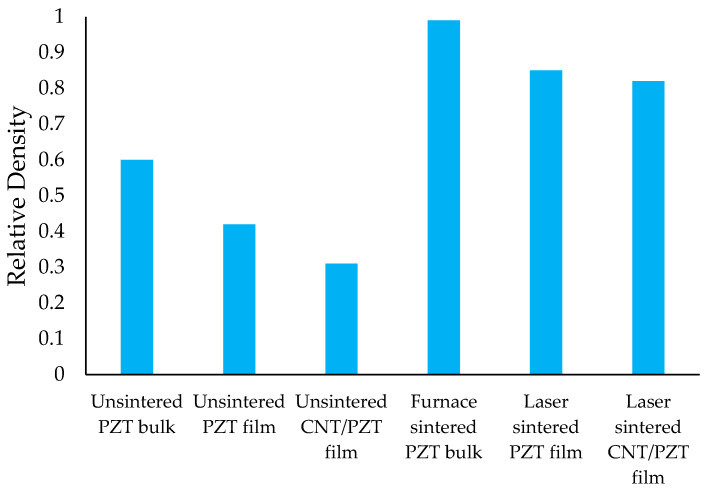
Relative density of the unsintered and sintered samples.

To estimate the density, the dimensions were obtained using a combination of contact measurement and non-contact optical profilometry, and the weight was measured using a digital balance. The laser-sintered films had thicknesses of 30–35 μm. The densities were presented as ratios to the PZT theoretical density of 7.9 g/cm^3^ [39] in Figure 12. During sintering, organic components were removed and the particles merged, resulting in densification and shrinkage of the thickness. The furnace produced a higher density compared with the laser. In addition to the long-duration heating, the densification in the bulk sample also benefitted from the pellet preparation process. It should be noted that the high pressure exerted by the hydraulic pressing machine on the pellet created a higher starting point for the bulk sample before sintering, compared to the slurry casting process. During laser sintering, the laser beam entered only from the top surface, and the high heating rate and short duration did not allow the whole sample to reach equilibrium. This caused uneven sintering in the transverse cross-section of the sample. Hence, the region near the top surface would have a higher density compared to the region near the bottom surface. The laser penetration factor, in addition to the lower density at starting point, resulted in lower sintered densities for the laser-processed films. For the sample containing CNTs, the density was further lowered. Although the absorption of CNT increased the temperature during sintering, CNT oxidation limited the densification. Furthermore, the density of CNTs is lower than PZT, which contributed to the lower overall density as well. In general, laser sintering created an appreciable increase in the density of the films compared with the unsintered state, which supported the enhancement of the piezoelectric property.

Mechanical testing was done using a microindenter. A Berkovich probe with a triangular pyramid geometry was lowered into the sample and a load was applied. The impression from the indentation and the load were measured. The following equation for the modulus of elasticity was applied:

(13) $$E=\frac{1-\nu^{2}}{\frac{2\beta \sqrt{A}}{S\sqrt{\pi }}-\frac{1-\nu_{i}^{2}}{E_{i}}}$$

where ν is Poisson’s ratio of PZT, which equals 0.31; β is a constant related to the geometry of the indenter, which equals 1.034 for the Berkovich probe used in this study [60]; A is the projected contact area; S is the maximum applied load; $\nu_{i}$ is Poisson’s ratio of the diamond indenter, which equals 0.07; and $E_{i}$ is the modulus of elasticity of the diamond indenter, which equals 1141 GPa. The hardness was obtained by dividing the maximum applied load by the contact area.

**Figure 13:**
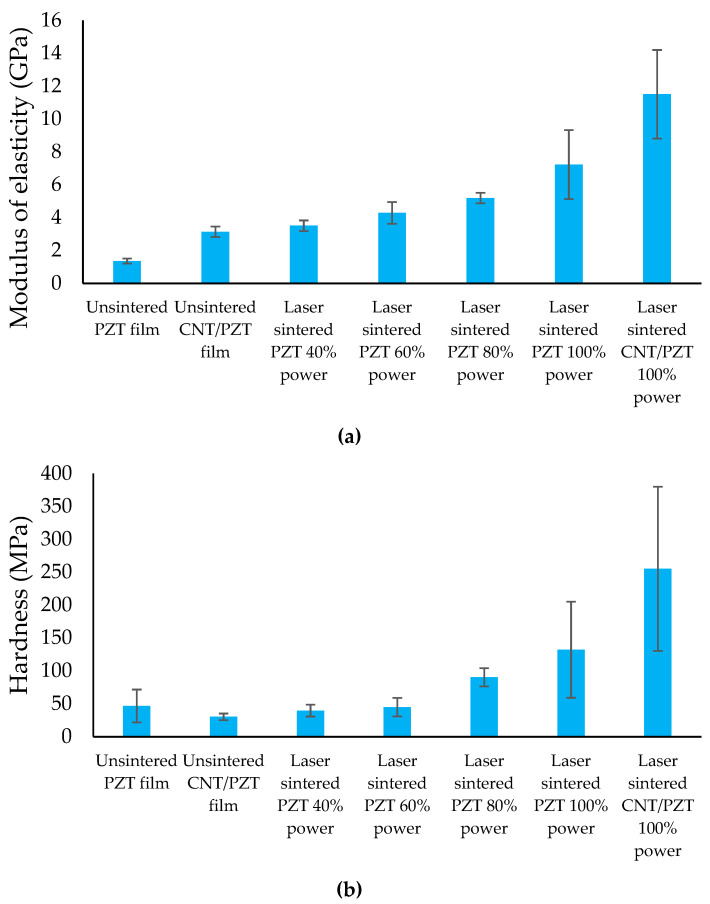
Mechanical properties of unsintered and laser sintered films. (**a**) Modulus of elasticity; (**b**) hardness.

The elastic modulus measurement results are presented in Figure 13a. Each sample was tested 10 times, and the standard deviation was calculated. This property had a positive correlation to the sintering power. After high-power sintering, the elastic modulus was much higher compared with the unsintered state. When CNTs were added, the elastic modulus increased. CNT possesses a very high modulus of elasticity, which contributed to the increase in the property of the composite. Although CNT oxidation occurred on the surface during the sintering, the reaction might not take place to completion, and the short duration could allow for a small amount of CNTs to remain, which acted as a reinforcement phase in the film. Figure 13b shows the hardness test results, including the standard deviations from 10 tests. The hardness followed the same general trend as the modulus of elasticity. This indicated that the effects of the rapid sintering method and the CNT had similar impacts on the hardness, where the high strength of CNT contributed to the property. The higher properties of the composite film made it more resistant to scratching, abrasion or fracturing, and thus, better able to maintain the integrity of the film.

### 4.5. Force-Sensing Application

**Figure 14:**
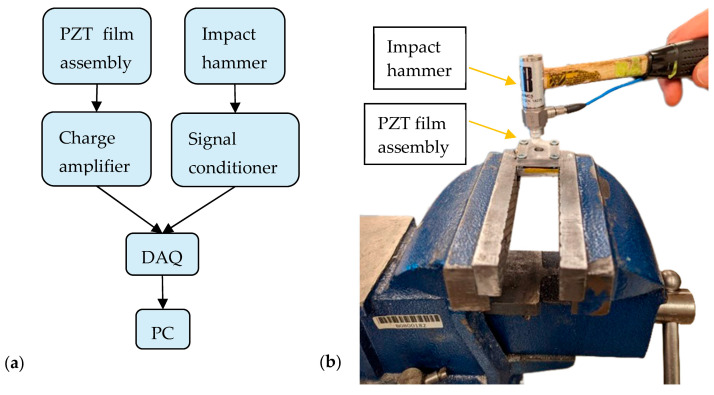
(**a**) PZT force sensor schematic; (**b**) photo of the testing process.

**Figure 15:**
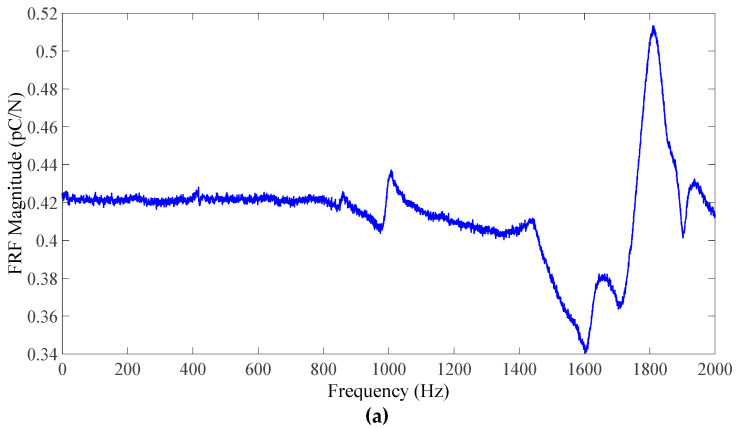

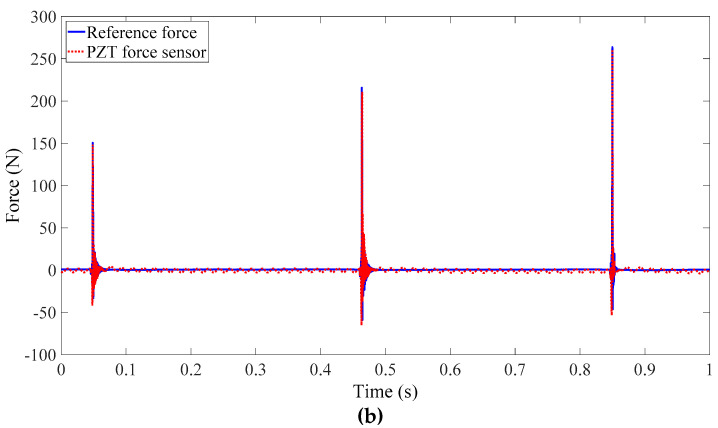
(**a**) Frequency response of the laser-sintered flexible PZT film–SS plate; (**b**) force response of the laser-sintered PZT film.

To compare the force-sensing application and adaptability with other low-cost and low-melting-point substrates, a composite sample was cast on an aluminum foil substrate and then laser sintered. In addition to demonstrating the adaptability, compared to the glass substrate, the Al foil would easily resist breakage during the hammer impact test, which was used to obtain the frequency response function of the sensor. Due to difference in thermal expansion and damage threshold with this substrate, the sample was laser sintered with adapted conditions, obtained by trial and error, i.e., at the focused position, 10% power and 15 μm line space. Figure 14 shows the force sensor developed using the laser-sintered sample. To characterize the response of the piezoelectric sensor, the PZT film was sandwiched between stainless steel (SS) plates and secured to a vice. The wires from the electrodes of the PZT film were connected to a charge amplifier (Kistler type 5010). The amplifier was routed to a data acquisition (DAQ) unit (National Instruments 9233), which was also interfaced with an impact hammer (PCB 208 A03) with a built-in sensor-amplifier circuit, and a PC. The impact hammer exerted a force in the same direction as the PZT polarity. The sensor was calibrated by applying one hit and comparing the signals from the hammer and the sensor. The frequency response function (FRF) of the sensing setup obtained by modal hammer tests is shown in Figure 15. It was subdued by the bolts used to secure the plates, but the setup provided a usable frequency range indication. The FRF indicates that the frequency linearity was maintained perfectly to around 850 Hz. Therefore the developed force sensor based on a laser-sintered PZT film is suitable for a wide frequency range.

After calibration, its performance was evaluated by applying multiple hits with different magnitudes. During the impact, the response signal immediately increased in relation to the force, and there was good linearity with the reference force, as shown in Figure 15. The usability of a laser-sintered PZT film on Al foil was demonstrated and other substrates and electrode patterns may be likewise explored with laser sintering.

### 4.6. Assumptions and Limitations

In the sintering model, the material properties of reflectance, thermal conductivity, heat capacity and emissivity were assumed to be independent of temperature. It was assumed that convective heat transfer was negligible, the ambient temperature was constant, and the material was homogeneous and isotropic. The assumptions made when applying the Beer–Lambert law were that the PZT medium was homogeneous, it did not scatter the radiation, the incident radiation consisted of parallel rays and it did not cause optical pumping.

In the fabrication, the process of manually adjusting the electrode tip prior to poling could produce a slight variation in the field strength. The piezoelectric meter also contained a measurement error of 5% [61]. The characterizations were conducted in a low-humidity environment; however, the electric resistance of the ceramic could decrease, which would cause a leakage current if the environment had a high humidity [62]. For the microindentation, the shape of the probe may have had an influence on the measurement.

The laser sintering was limited by the level of densification to avoid damage near the substrate interface. Hence, the piezoelectric and mechanical properties were lower than the fully dense sintering from the furnace. The properties were nevertheless largely retained during the laser processing and can be utilized for application.

## 5. Conclusions

Sintering using a laser was demonstrated to obtain piezoelectric ceramic film via a low-cost alternative method. The time and energy used to fabricate the laser-sintered sample was a small fraction compared with conventional furnace sintering. Compared with dozens of hours of sintering time often required and multiples of kilowatts used in the thermal furnace method, the laser method reduced the sintering time to a couple of hours including preheating, and the power usage to approximately 450 watts maximum, including the preheating, laser source and PC setup. The laser setup is adaptable to facilitate low-cost mass production. It was confirmed that the temperature limitations of the different materials can be overcome in order to deploy the flexible substrates. Although the overall density of the PZT film sintered using a laser was lower compared with the conventional furnace method, the piezoelectric response still showed high sensitivity. Moreover, adding CNTs to create a composite increased the hardness and strength while largely retaining the piezoelectric property and using less energy for sintering. In contrast to the furnace method, whose extended sintering period causes excessive oxidation of the composite sample, the short pulses of the laser method are compatible with sintering the composite. The composite device will be suitable for more environments that require more durability.

Models of the laser sintering environment were developed to build an understanding of the laser interaction inside the material and the sintering phenomenon. The models were used to support and verify the investigation. The high power density required a careful selection of laser parameters. Optimization of the laser parameters focused on allowing for the elimination of the organic binder, avoiding degradation of the perovskite structure and substrate, avoiding crack or porosity formation, and improving the sintering consistency across the surface. The film was used in a sensing setup, in which the signal from the film was interpreted to obtain the force. Future applications could be tightly integrated sensors for measuring force due to the high-frequency response and minimization of the impact on the dynamics.

## Figures and Tables

**Table 1:** Composition of the slurry.

Materials	Weight Percentage for PZT Samples	Weight Percentage for PZT/CNT Composite Samples
PZT powder	64.025	63.7
Triethyl phosphate	0.975	0.975
Carbon nanotube	0	0.325
α-Terpineol	34.3	34.3
Ethyl cellulose	0.7	0.7

**Table 2:** Material properties of PZT and substrate used in the finite element simulation.

Material	Property	Value	Unit
PZT	Refractive index (n)	2.38 [34]	
	Extinction coefficient (k)	0.05 at T < 1050 °C [34] 0.001 at T > 1050 °C [35,36]	
	Thermal conductivity (kt)	1.3 [38]	W/(m·K)
	Heat capacity (Cp)	440 [38]	J/(kg·K)
	Density (ρ)	3300 at T < 1400 °C 7900 at T > 1400 °C [39]	kg/m^3^
	Thickness	45	μm
	Emissivity (ε)	0.9 [40]	
Indium tin oxide	Refractive index (n)	1.59	
	Extinction coefficient (k)	0.0057 [41]	
	Thermal conductivity (kt)	5.86 [42]	W/(m·K)
	Heat capacity (Cp)	341 [43]	J/(kg·K)
	Density (ρ)	7120 [44]	kg/m^3^
	Thickness	185 [45]	nm
Soda-lime glass	Refractive index (n)	1.5129	
	Extinction coefficient (k)	4.9238 × 10^−6^ [46]	
	Thermal conductivity (kt)	1 [47]	W/(m·K)
	Heat capacity (Cp)	800 [48]	J/(kg·K)
	Density (ρ)	2.53 [49]	kg/m^3^
	Thickness	1.1	mm

**Table 3:** Laser setup properties used in the finite element simulation [50,51].

Property	Value	Unit
Beam quality factor (M^2^)	1.4	
Centre wavelength (λ)	1064	nm
Pulse energy (maximum) (E)	0.68	mJ
Pulse duration (FWHM) (τ)	100	ns
Repetition rate	30	kHz
Beam diameter (1/e^2^) (D)	7	mm
Focus length (f)	254	mm

**Table 4:** Material properties of CNT/PZT used in the finite element simulation.

Property	Value (0.5% CNTs)	Unit
n	2.37	
k	0.58679 at T < 1050 °C 0.01133 at T > 1050 °C	
Thermal conductivity	3.78	W/(m·K)
Heat capacity	328	J/(kg·K)
Density	3300 at T < 1400 °C 7900 at T > 1400 °C	kg/m^3^
Emissivity	0.90	

**Table 5:** Piezoelectric coefficient comparison.

Sintering Method and Composition	Unsintered PZT	Furnace-Sintered PZT Bulk	Furnace-Sintered PZT Film	Laser-Sintered PZT	Laser-Sintered CNT/PZT
$d_{33}$ **(pC/N)**	7	420	130	67	42

**Table 6:** Piezoelectric coefficient of PZT laser sintered using 40% to 100% power.

Laser Power	40%	60%	80%	100%
$d_{33}$ **(pC/N)**	48	50	54	67

**Table 7:** Average peak-to-valley measurement comparison of sintered samples.

Sintering Method and Composition	Furnace-Sintered PZT	Laser-Sintered PZT	Laser-Sintered CNT/PZT
Sz **(μm)**	0.886	1.329	2.311

## Data Availability

Not applicable.

## References

[1] Rao S.S. (2011). Mechanical Vibrations.

[2] Gao W., Zhu Y., Wang Y., Yuan G., Liu J.-M. (2020). A review of flexible perovskite oxide ferroelectric films and their application. J. Mater..

[3] Callister W.D., Rethwisch D.G. (2018). Materials Science and Engineering: An Introduction.

[4] Heywang W., Lubitz K., Wersing W. (2008). Piezoelectricity: Evolution and Future of a Technology.

[5] Park K.-I., Son J.H., Hwang G.-T., Jeong C.K., Ryu J., Koo M., Choi I., Lee S.H., Byun M., Wang Z.L. (2014). Highly-Efficient, Flexible Piezoelectric PZT Thin Film Nanogenerator on Plastic Substrates. Adv. Mater..

[6] Wu W., Bai S., Yuan M., Qin Y., Wang Z.L., Jing T. (2012). Lead Zirconate Titanate Nanowire Textile Nanogenerator for Wearable Energy-Harvesting and Self-Powered Devices. ACS Nano.

[7] Qi Y., Kim J., Nguyen T.D., Lisko B., Purohit P.K., McAlpine M.C. (2011). Enhanced Piezoelectricity and Stretchability in Energy Harvesting Devices Fabricated from Buckled PZT Ribbons. Nano Lett..

[8] Kang M.-G., Noh M.-S., Pyeon J.J., Jung W.-S., Moon H.G., Baek S.-H., Nahm S., Yoon S.-J., Kang C.-Y. (2020). Direct Growth of Ferroelectric Oxide Thin Films on Polymers through Laser-Induced Low-Temperature Liquid-Phase Crystallization. Chem. Mater..

[9] Song L., Glinsek S., Defay E. (2021). Toward Low-Temperature Processing of Lead Zirconate Titanate Thin Films: Advances, Strategies, and Applications. Appl. Phys. Rev..

[10] Yu M.-F., Lourie O., Dyer M.J., Moloni K., Kelly T.F., Ruoff R.S. (2000). Strength and Breaking Mechanism of Multiwalled Carbon Nanotubes under Tensile Load. Science.

[11] Berber S., Kwon Y.-K., Tománek D. (2000). Unusually High Thermal Conductivity of Carbon Nanotubes. Phys. Rev. Lett..

[12] Huang Q., Gao L. (2004). Manufacture and Electrical Properties of Multiwalled Carbon Nanotube/BaTiO_3_ Nanocomposite Ceramics. J. Mater. Chem..

[13] Jia Y., Ajayi T.D., Morales J., Chowdhury M.A.R., Sauti G., Chu S.-H., Park C., Xu C. (2019). Thermal Properties of Polymer-Derived Ceramic Reinforced with Boron Nitride Nanotubes. J. Am. Ceram. Soc..

[14] Rezvani S., Chuo Y.S., Lee J., Park S.S. (2022). Hybrid Sintering of CNT/PZT Ceramics Using Microwave Oven. Ceram. Int..

[15] Cox J., Woods B., Hertel M., Peters E. Graphite Oxidation Rates in Comparison to Regimes with Application to the Oregon State University High Temperature Test Facility. Proceedings of the International Topical Meeting on Nuclear Reactor Thermal Hydraulics.

[16] Bharadwaja S.S.N., Griggio F., Kulik J., Trolier-McKinstry S. (2011). Highly Textured Laser Annealed Pb(Zr_0.52_Ti_0.48_)O_3_ Thin Films. Appl. Phys. Lett..

[17] Chen X., Yagi M. (2014). Development of crystallization of PZT films by laser annealing. Ricoh Tech. Rep..

[18] Rajashekhar A., Fox A., Bharadwaja S.S.N., Trolier-McKinstry S. (2013). In Situ Laser Annealing during Growth of Pb(Zr_0.52_Ti_0.48_)O_3_ Thin Films. Appl. Phys. Lett..

[19] Schatz A., Pantel D., Hanemann T. (2017). Pulsed Laser Deposition of Piezoelectric Lead Zirconate Titanate Thin Films Maintaining a Post-CMOS Compatible Thermal Budget. J. Appl. Phys..

[20] Bernard S.A., Balla V.K., Bose S., Bandyopadhyay A. (2010). Direct Laser Processing of Bulk Lead Zirconate Titanate Ceramics. Mater. Sci. Eng. B.

[21] Tarasova E., Juravleva I., Shishkovsky I., Ruzhechko R. (2013). Layering Laser-Assisted Sintering of Functional Graded Porous PZT Ceramoplasts. Phase Transit..

[22] Mistler R.E., Twiname E.R. (2000). Tape Casting: Theory and Practice.

[23] Huang X., Raether F. (2009). Role of Impurities in the Sintering Behavior and Properties of Lead Zirconate Titanate Ceramics. J. Am. Ceram. Soc..

[24] Carbon Nanotubes History And Production Methods. https://www.cheaptubes.com/.

[25] Rezvani S., Kim C.-J., Park S.S., Lee J. (2020). Simultaneous Clamping and Cutting Force Measurements with Built-in Sensors. Sensors.

[26] Waller D., Safari A. (1988). Corona Poling of PZT Ceramics and Flexible Piezoelectric Composites. Ferroelectrics.

[27] Hecht E. (2017). Optics.

[28] Carniglia C.K., Apfel J.H. (1980). Maximum Reflectance of Multilayer Dielectric Mirrors in the Presence of Slight Absorption. J. Opt. Soc. Am..

[29] What Is A F-Theta Len?. https://www.galvo-scanner.com/info/what-is-a-f-theta-len-49672252.html.

[30] Sun H. (2015). A Practical Guide to Handling Laser Diode Beams.

[31] Hill D. Ansys Zemax Knowledgebase: How to Convert FWHM Measurements to 1/(e2) Halfwidths. https://support.zemax.com/hc/en-us/articles/1500005488161.

[32] Weisstein E.W. Gaussian Function. https://mathworld.wolfram.com/.

[33] Donohue P.P., Todd M.A. (2000). Pulse-Extended Excimer Laser Annealing of Lead Zirconate Titanate Thin Films. Integr. Ferroelectr..

[34] Khodorov A., Gomes M.J.M. (2006). Preparation and Optical Characterization of Lanthanum Modified Lead Zirconate Titanate Thin Films on Indium-Doped Tin Oxide-Coated Glass Substrate. Thin Solid Film..

[35] Ban D., Liu G., Yu H., Sun X., Deng N., Qiu F. (2021). High Electro-Optic Coefficient Lead Zirconate Titanate Films toward Low-Power and Compact Modulators. Opt. Mater. Express.

[36] Tang X.G., Liu Q.X., Jiang L.L., Ding A.L. (2007). Optical Properties of Pb(Zr_x_Ti_1−x_)O_3_ (X = 0.4, 0.6) Thin Films on Pt-Coated Si Substrates Studied by Spectroscopic Ellipsometry. Mater. Chem. Phys..

[37] Peck E.R., Reeder K. (1972). Dispersion of Air. J. Opt. Soc. Am..

[38] Manion D. (2020). Materials Technical Data (Typical Values).

[39] TCERA Co (2019). TCERA Piezoelectric Material, Characteristics.

[40] Trebla Services (2012). Emissivity Table.

[41] Moerland R., Hoogenboom J.P. (2016). Complex Refractive Index of ITO (Indium Tin Oxide)—ITO Cover Slides from Optics Balzers.

[42] Ashida T., Miyamura A., Oka N., Sato Y., Yagi T., Taketoshi N., Baba T., Shigesato Y. (2009). Thermal Transport Properties of Polycrystalline Tin-Doped Indium Oxide Films. J. Appl. Phys..

[43] Wang L., Wen J., Yang C., Xiong B. (2018). Potential of ITO Thin Film for Electrical Probe Memory Applications. Sci. Technol. Adv. Mater..

[44] Wypych G. (2014). Databook of Antistatics.

[45] 1.1 mm 7~10 Ohm/Sq ITO Coated Glass Substrate. https://www.msesupplies.com/products/7-9-ohm-sq-ito-substrate-indium-doped-tin-oxide-in-sno2-or-ito-coated-glass-can-customize-conductive-film-patterns-as-required.

[46] Rubin M. (1985). Optical Properties of Soda Lime Silica Glasses. Sol. Energy Mater..

[47] Janssen L.P.B.M., Warmoeskerken M.M.C.G. (2006). Transport Phenomena Data Companion.

[48] Soda-Lime (Float) Glass. https://www.makeitfrom.com/material-properties/Soda-Lime-Float-Glass.

[49] Seward T.P., Vascott T. (2005). High Temperature Glass Melt Property Database for Process Modeling.

[50] Max Photonics Co. (2020). MFP 10W~70W Q-Switch Pulsed Fiber Laser.

[51] Shenzhen JGZ Optical Technology Co. (2019). 1064nm F-Theta Lens.

[52] Ermolaev G.A., Tsapenko A.P., Volkov V.S., Anisimov A.S., Gladush Y.G., Nasibulin A.G. (2020). Express Determination of Thickness and Dielectric Function of Single-Walled Carbon Nanotube Films. Appl. Phys. Lett..

[53] Pradhan N.R., Duan H., Liang J., Iannacchione G.S. (2009). The Specific Heat and Effective Thermal Conductivity of Composites Containing Single-Wall and Multi-Wall Carbon Nanotubes. Nanotechnology.

[54] Aktakka E.E., Peterson R.L., Najafi K. (2013). Wafer-Level Integration of High-Quality Bulk Piezoelectric Ceramics on Silicon. IEEE Trans. Electron Devices.

[55] Tadigadapa S. (2010). Piezoelectric Microelectromechanical Systems—Challenges and Opportunities. Procedia Eng..

[56] Piezotech Co. (2018). TDS Piezotech FC..

[57] Rouquette J., Haines J., Bornand V., Pintard M., Papet P., Bousquet C., Konczewicz L., Gorelli F.A., Hull S. (2004). Pressure Tuning of the Morphotropic Phase Boundary in Piezoelectric Lead Zirconate Titanate. Phys. Rev. B.

[58] Chandran M., Tiwari B., Kumaran C.R., Samji S.K., Bhattacharya S.S., Rao M.S.R. (2012). Integration of Perovskite PZT Thin Films on Diamond Substrate without Buffer Layer. J. Phys. D Appl. Phys..

[59] Ouyang J., Cormier D., Williams S.A., Borkholder D.A. (2016). Photonic Sintering of Aerosol Jet Printed Lead Zirconate Titanate (PZT) Thick Films. J. Am. Ceram. Soc..

[60] Oliver W.C., Pharr G.M. (1992). An Improved Technique for Determining Hardness and Elastic Modulus Using Load and Displacement Sensing Indentation Experiments. J. Mater. Res..

[61] Cable T. (2021). APC International, Ltd. Piezo d_33_ Test System.

[62] Zheng D., Swingler J., Weaver P. (2010). Current Leakage and Transients in Ferroelectric Ceramics under High Humidity Conditions. Sens. Actuators A Phys..

